# Embryonic spatiotemporal expression pattern of Folded gastrulation suggests roles in multiple morphogenetic events and regulation by AbdA

**DOI:** 10.1093/g3journal/jkae032

**Published:** 2024-02-15

**Authors:** Vrushali Katagade, Manisha Kandroo, Anuradha Ratnaparkhi

**Affiliations:** MACS-Agharkar Research Institute (Affiliated to Savitribai Phule Pune University), Developmental Biology Group, G.G. Agarkar Road, Pune 411 004, Maharashtra, India; MACS-Agharkar Research Institute (Affiliated to Savitribai Phule Pune University), Developmental Biology Group, G.G. Agarkar Road, Pune 411 004, Maharashtra, India; MACS-Agharkar Research Institute (Affiliated to Savitribai Phule Pune University), Developmental Biology Group, G.G. Agarkar Road, Pune 411 004, Maharashtra, India

**Keywords:** Folded gastrulation, Fog, AbdA, morphogenesis, glia

## Abstract

In *Drosophila*, the signaling pathway activated by the ligand Folded gastrulation (Fog) is among the few known G protein–coupled receptor (GPCR) pathways to regulate cell shape change with a well-characterized role in gastrulation. However, an understanding of the spectrum of morphogenetic events regulated by Fog signaling is still lacking. Here, we present an analysis of the expression pattern and regulation of *fog* using a genome-engineered Fog::sfGFP line. We show that Fog expression is widespread and in tissues previously not associated with the signaling pathway including germ cells, trachea, and amnioserosa. In the central nervous system (CNS), we find that the ligand is expressed in multiple types of glia indicating a prominent role in the development of these cells. Consistent with this, we have identified 3 intronic enhancers whose expression in the CNS overlaps with Fog::sfGFP. Further, we show that *enhancer-1*, (*fogint^enh-1^*) located proximal to the coding exon is responsive to AbdA. Supporting this, we find that *fog* expression is downregulated in *abdA* mutants. Together, our study highlights the broad scope of Fog-GPCR signaling during embryogenesis and identifies Hox gene AbdA as a novel regulator of *fog* expression.

## Introduction

Mechanisms that regulate cell shape change play a critical role during morphogenesis—a process that underlies formation of 3D organs and tissue structures. Shape and geometry influence not just how cells organize themselves with respect to their neighbors but also cell–cell signaling and differentiation, by controlling the cellular or subcellular distance between the signal source and target (Sordella *et al.* 2003; McBeath *et al.* 2004; Nelson *et al.* 2005; Meyers *et al.* 2006; Gao *et al.* 2010). Outside the developmental context, processes such as wound healing, migration of immune cells, or tumor cells during metastasis, also involve extensive cell shape change. Further, in many neurodegenerative disorders, “reactive astrocytes” that have altered morphology play a key role in disease progression (Schiweck *et al.* 2018; Sofroniew 2020; Patani *et al.* 2023).

Signaling pathways that trigger change in cell shape do so through reorganization of the cellular cytoskeleton that generates the necessary mechanical force essential for this process. Actin, together with myosin, is a key generator of this force and therefore a convergence point for these pathways (reviewed in Paluch and Heisenberg 2009; Martin 2010; Perez-Vale and Peifer 2020).

Gastrulation in *Drosophila* has been used extensively as a model to understand the molecular, cellular, and biophysical mechanisms that drive cell shape change essential for tissue bending, folding, and extension. Among these, apical constriction is a form of change in which the apical end of an epithelial cell contracts to reduce its surface area while simultaneously broadening its basal side resulting in a wedge-shaped cell. This type of shape change is seen in epithelial sheets during bending, folding, and tube formation (Sawyer *et al.* 2010; Martin 2010; Martin and Goldstein 2014). The process involves nonmuscle myosin II (hereafter referred to as myosin) dependent contraction of the actin cytoskeleton at the apical end while maintaining cell–cell adhesion. This is an active process and involves dynamic assembly and reassembly of actin (Jodoin *et al.* 2015)

The G protein–coupled receptor (GPCR) signaling pathway triggered by ligand Folded gastrulation (Fog) is a key regulator of apical constriction during gastrulation (Costa *et al.* 1994). GPCRs encoded by genes *mist* and *smog* have been identified as transducers of the Fog signal (Manning *et al.* 2013; Manning and Rogers 2014; Kerridge *et al.* 2016), and activation of this pathway leads to relocalization of basal myosin to the apical side, to facilitate constriction (Dawes-Hoang *et al.* 2005).

The G protein that mediates Fog signaling in this context is encoded by *concertina* which belongs to the Gα_12/13_ family of G proteins. In vertebrates, this family of G proteins regulates a variety of physiological processes including cell migration and actin dynamics (Parks and Wieschaus 1991; Morize *et al.* 1998; Yang *et al.* 2020; Guo *et al.* 2022). Concertina signals to downstream RhoGEF2 leading to changes in the actin cytoskeleton (Barrett *et al.* 1997; Häcker and Perrimon 1998; Morize *et al.* 1998).

Fog signaling has also been implicated in the morphogenesis of the salivary glands (SGs) and wing imaginal discs (Lammel and Saumweber 2000; Nikolaidou and Barrett 2004; Chung *et al.* 2017; Vishwakarma *et al.* 2022); in the embryonic central nervous system (CNS), loss of *fog* leads to defects in motor axon guidance and glial morphogenesis (Ratnaparkhi and Zinn 2007).

The role of GPCR signaling in regulating morphogenesis, particularly during development, has not been studied extensively, and Fog signaling is among the few known to operate during development. However, the extent to which this pathway influences morphogenesis during development, and its role in other physiological processes if any, remains relatively unexplored. This is partly due to the lack of a comprehensive understanding of the localization and spatiotemporal expression pattern of Fog at different developmental stages. In this study, we have sought to address this by examining tissue-specific localization of Fog using a Fog::sfGFP (superfolder GFP) line in which endogenous *fog* is tagged with sfGFP at the C-terminal end of the protein using CRISPR technology.

We find that expression of Fog::sfGFP in the embryo is widespread and present tissues that, thus far, have not been associated with Fog signaling. This includes the primordial germ cells (PGCs), amnioserosa, and embryonic epidermis. In the CNS, we detect expression of the ligand in multiple types of glia suggesting that it may play a role in the development and morphogenesis of these cells. This latter observation is supported by our analysis of 3 intronic enhancers whose expression pattern in glia overlaps significantly with Fog::sfGFP.

We find that of the 3 enhancers, the proximal element (*fogint^enh-1^*) drives reporter expression in the ventral furrow during gastrulation and in cell body glia (CBG), with expression restricted only to glia in the abdominal segments.

Based on this expression pattern, we have sought to test whether the enhancer is responsive to Hox protein AbdA. We have conducted in silico analysis to identify Hox-binding sites and used gain-of-function analyses to show that this *cis*-regulatory element is responsive to AbdA. Using a converse approach, we show that *abda* mutants have reduced *fog* expression at the ventral furrow indicating a role for AbdA in regulating *fog* expression.

Together, our study highlights the broad scope of Fog-GPCR signaling during development and its prominent role in the CNS and identifies Hox gene *abdA* as a novel regulator of *fog* expression.

## Materials and methods

### Generation of Fog::sfGFP

Generation of Fog::sfGFP was carried out at the fly facility, C-CAMP, Bengaluru. CRISPR-based homologous recombination (HR) strategy was used to generate fly lines expressing Fog protein fused at its C-terminus to sfGFP. Guide RNA (gRNA) target (GCAGCAGCAACTCCTGGCTCTGG) was selected in the 3′UTR, 20 bases downstream of the stop codon. gRNA was cloned in pBFVU6.2 vector (Kondo and Ueda 2013) using a standard PCR-based strategy. HR construct was generated by Gibson assembly using pHD-sfGFP-ScarlessDsRed vector (DGRC Stock 1365; https://dgrc.bio.indiana.edu//stock/1365; RRID:DGRC1365) such that the C-terminus of *fog* is in frame with sfGFP. Since the 3′UTR was left intact in the HR construct, site-directed mutagenesis was performed to remove the PAM site to ensure that the gRNA target was absent from this construct.

To generate knock-in flies, ∼500 embryos from flies expressing *vasa*-Cas9 on the third chromosome (BDSC#51324) were injected with gRNA (250 ng/µl) and HR construct (750 ng/µl) using standard microinjection protocol using Eppendorf FemtoJet. Surviving adults (207) were self-crossed and F1 progeny was screened for the presence of 3XP3-dsRed marker in the eyes. Eight positive lines were recovered, out of which 6 were found to be correctly localized. These were then made into homozygous stocks.

### Generation of reporter lines using intronic enhancer sequences

Primers were designed to intronic regions present in the intron immediately 5′ to the coding exon. These regions were PCR amplified from genomic DNA isolated from *w^1118^* larvae and cloned directionally into the D-TOPO vector using the manufacturer's instructions (pENTR/D-TOPO Kit, Thermo Fisher, Cat. No K240020SP). Clones were confirmed first through restriction digest and later sequencing. Gateway cloning using LR clonase was used to insert the enhancer fragments into destination vectors pDESTHemmar-G or pDESTHemmar-R (Han *et al.* 2011). PCR was used confirm cloning. Injection and generation of transgenics was carried out by the fly facility, C-CAMP, Bengaluru. The primer sequences used for cloning the different enhancers are listed below:

- *fog^int-enh1^*
  Fwd primer: 5′ ATGCATTGCAGCATGCGTC 3′
  Rev Primer: 5′ AGAACAGCCAGCAGACAATTG 3′
- *fog^int-enh2^*
  Fwd primer: 5′ ATACGTGTCGACACGAAGC 3′
  Rev Primer: 5′ GACGCATGCTGCAATGCA 3′
- *fog^int-enh3^*
  Fwd primer: 5′ GCACTCGACTCACACTCAAC 3′
  Rev Primer: 5′ GCTTCGTGTCGACACGTAT 3′

#### Fly husbandry

*fog^int-enh1^*::RFP, *fog^int-enh2^*::GFP, and *fog^int-enh3^*::GFP transgenic lines were generated as part of this study as described above. *fog^int-enh1^*::RFP and *fog^int-enh3^*::GFP carry the transgene on the second chromosome; *fog^int-enh2^*::GFP carries the insertion on the third chromosome. Expression of Fog::sfGFP compared across multiple lines showed identical patterns of expression. All data in this manuscript are derived from line 3.

Other fly lines used in this study include *w^1118^*, *UAS-abdA* (2nd Chr, Lelli *et al.* 2011); *repo*-GAL4 (IInd chromosome); *abda^MX1/MX1^* (kind gift from Technau Lab); and *UAS-fogRNAi* (Ratnaparkhi and Zinn 2007). The RNAi and overexpression experiments were conducted at 25°C. Recombinant lines used in this study were generated using standard genetic techniques.

#### Immunohistochemistry, imaging, and image analysis

Embryos were fixed and stained using standard procedures described in Shweta *et al.* 2021. Larval brains were dissected in PBS and fixed with 4% paraformaldehyde for 20 min followed by 3–4 washes with PBS for 15 min to remove traces of fixative. The subsequent steps for antibody staining were similar to those used for embryos. Tissues were mounted in vectashield (Vector Labs) to prevent fading. The following antibodies were used: anti-GFP (1:200, Cat. No: A10262, Thermo Fisher), anti-DE-Cad (1:20; Dcad2, DSHB, Iowa), anti-Futsch (1:25; 22C10, DSHB, Iowa), anti-Repo (1:25; 8D12, DSHB, Iowa), anti-coracle (1:50; C566.9, DSHB, Iowa), anti-digoxigenin (DIG) rhodamine (1:500; Cat. No: 1133089001, Roche), anti-DIG alkaline phosphatase (AP; 1:500; Cat. No: 11093274910, Roche), anti-beta-galactosidase (1:500; Cat. No: Z3781, Promega), anti-beta-galactosidase (1:10; Cat. No: 40-1a, DSHB, Iowa), anti-RFP (1:200, Cat. No: AB62341, Abcam), and anti-Prospero (Pros) (1:20, MR1A, DSHB, Iowa).

**Fig. 1.**
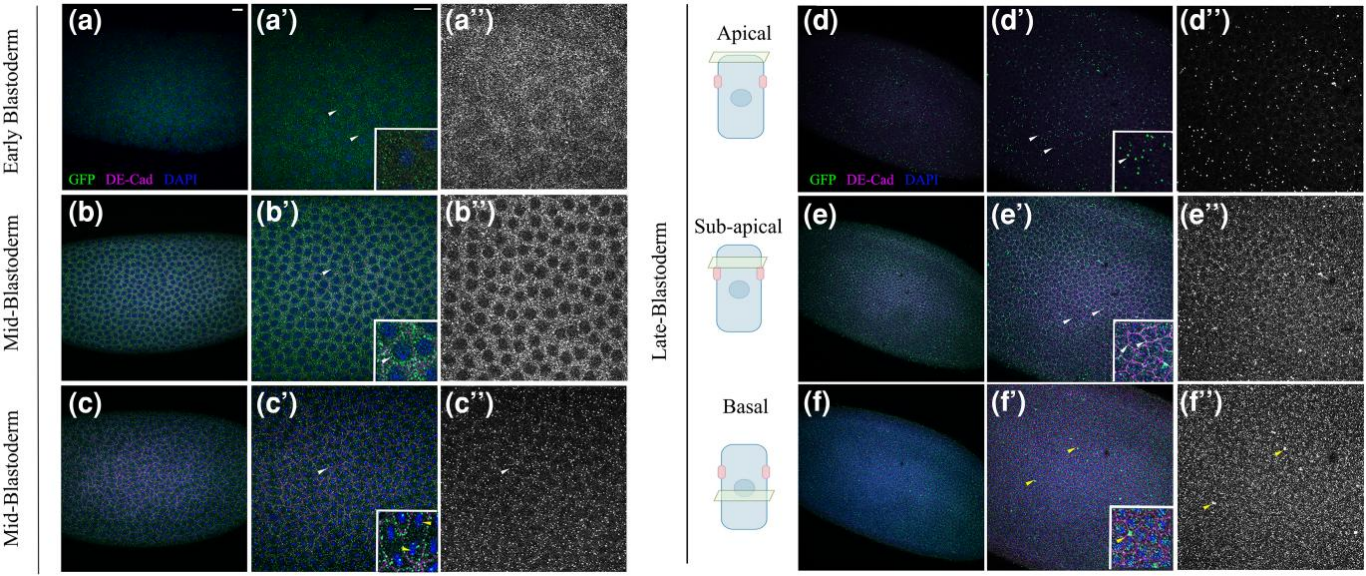
Expression pattern and localization of Fog::sfGFP in blastoderm embryos. a–a″) Localization of Fog::sfGFP in early syncytial blastoderm. Punctate expression (arrowheads) is detected around nuclei. b–b″) Syncytial embryo at mid-blastoderm stage. Punctate GFP staining is seen along the lateral membrane (arrowhead). c–c″) Syncytial embryo undergoing nuclear division. Fog::sfGFP localizes along adherens junctions marked by DE-Cadherin. d, f) Localization of Fog::sfGFP along the apico-basal axis in late blastoderm embryo. d–d″) Apical sections show randomly distributed Fog::sfGFP puncta (white arrowhead); e–e″) Subapical region shows presence of Fog::sfGFP along adherens junctions regions marked by DE-Cadherin (see inset, arrowhead). f, f″) Basal region shows medial localization of Fog::sfGFP (inset, white arrowhead); arrowhead in f'-f'' indicates the occasional accumulation of the protein at cell–cell junctions. Scale bar is 20 μm.

Images were obtained using Zeiss LSM 900 and Leica SP800 confocal system with a 40× oil objective (0.95 NA). Embryos imaged using Zeiss LSM900 were taken at 0.45× for maximal tissue coverage. Respective zoom images were taken at 0.75× or 1×. Images of the ventral nerve cord (VNC) shown in Fig. 5c and d were taken using a 63× oil objective (1.4 NA). Biorender (https://www.biorender.com) was used for generating drawings shown in Fig. 1 and Fig. 7A.

Fluorescence intensity was measured using the methodology described by Menon *et al.* (2009). Generally, embryos at 15 or 16 were used for analysis. Care was taken to ensure that the numbers and stages represented in control and experimental sets were similar. Briefly, using FIJI/ImageJ, we first generated a sum projection of the “z” sections that encompass the CBG and measured the mean gray value for segments A1–A7. Background signal for the same area is measured from the adjacent region (outside the CNS) and subtracted from the signal to obtain the mean intensity value for that embryo. In the graph, each dot represents the normalized intensity value for 1 embryo. Student's *t*-test was used to measure statistical significance.

### RNA in situ hybridization

RNA in situ hybridization on 0- to 4-h wild-type and *abdA^MX1/MX1^* mutant embryos was carried out using sense and antisense DIG-labeled RNA probes against *fog* using the method described in Patel (1994). Homozygous *abdA* mutants were identified based on the absence of TM3*ftzlacZ* balancer that was detected by staining the embryos with anti-beta-galactosidase antibody. Probe detection was carried out using anti-DIG antibody tagged with rhodamine or alkaline phosphatase. In case of the latter, NBT/BCIP was used as substrate for the color reaction. Imaging of fluorescently stained embryos was carried out on Leica SP8 system, 40× oil objective (NA 1.3).

## Results

### Fog shows differential localization in the blastoderm embryo

*Fog* mRNA is maternally deposited into the embryo and uniformly distributed prior to cellularization. Zygotic expression is turned on at the start of cellularization leading to strong localized expression in the ventral and posterior regions of the embryo (Costa *et al.* 1994). As one of the first steps toward validating our CRISPR line, we examined the expression and localization of Fog::sfGFP in early embryos at blastoderm and gastrulation. Consistent with the observations by Costa *et al.* (1994), we could detect Fog::sfGFP expression in early preblastoderm embryos. At this stage, Fog::sfGFP puncta were uniformly distributed around the nuclei present in the middle of the embryo; expression of DE-Cadherin was not observed at this stage (Fig. 1a–a″). At the syncytial mid-blastoderm stage, Fog::sfGFP expression appeared more prominent and was present along the lateral margins defined by DE-Cadherin (Fig. 1b–b″). This pattern of localization along the lateral margin could be observed even during nuclear division (Fig. 1c and c″).

Interestingly, in late blastoderm embryos, we observed differential localization of Fog along the apico-basal axis: In the apical most sections, Fog::sfGFP appeared randomly distributed throughout the blastoderm (Fig. 1d–d″). Most of these appeared to be medially situated. In the subapical region, expression was predominantly along the lateral edges marked by DE-Cadherin. A few discrete GFP-positive puncta could be seen located medially (Fig. 1e–e″). However, in the basal region, these puncta were present medially throughout the cellular space.

**Fig. 2.**
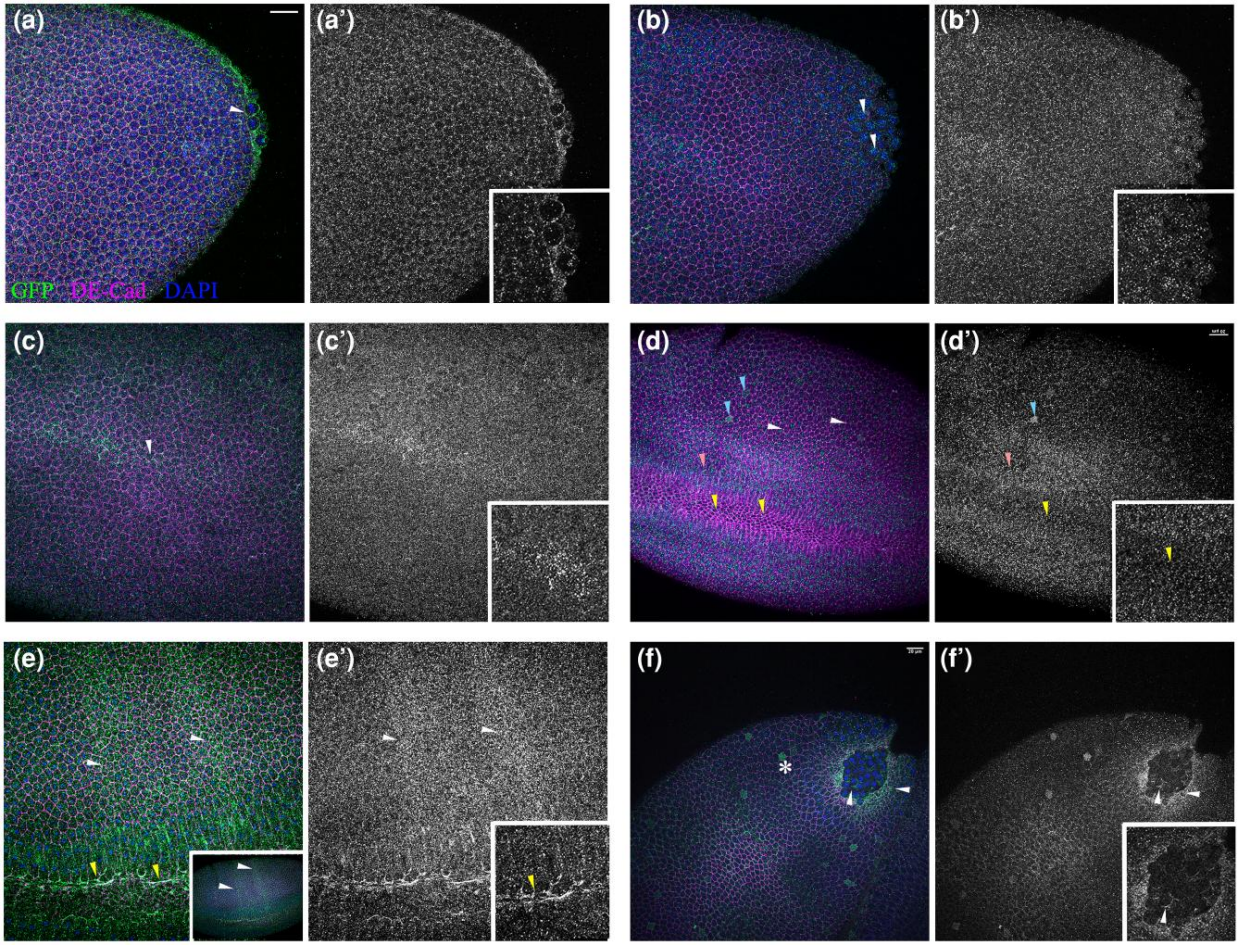
Fog::sfGFP expression in the epidermis and pole cells of blastoderm embryos. a, b) Fog::sfGFP expression in pole cells (color and gray scale) of a stage 10 blastoderm embryo (arrowhead). c,c')– Apical expression of Fog::sfGFP in cells of the presumptive mesoderm prior to formation of the ventral furrow. d, d') GFP staining in the ventral furrow indicated by yellow arrowheads. Basal expression in cells flanking the furrow is indicated by pink arrowheads; “islands” of Fog::sfGFP in the lateral epidermis is marked by blue arrowheads; (e, e') Striped expression of Fog::sfGFP along the D-V axis at gastrulation. Inset shows expression in the ventral furrow. f, f′) Expression of Fog::sfGFP at the posterior midgut primordium and in pole cells (arrowhead). Inset shows expression in pole cells. Asterisk indicates ‘islands’ of Fog::sfGFP expression. Scale bar is 20 μm.

Unexpectedly, we found GFP expression in budding pole cells (Fig. 2a–a″, b, and b′). The staining was also observed in embryos obtained from a second-independent Fog::sfGFP line making it unlikely to be an artifact of immunostaining. Together, these results suggest a role for Fog signaling in pre and early blastoderm embryos, prior to the onset of gastrulation, and this function is likely to be regulated by the maternal component of the gene.

We next examined the expression of Fog::sfGFP during gastrulation. The earliest expression that we could detect on the ventral side was towards the end of cellularization and prior to the formation of the ventral furrow. At this stage, expression was apical and asymmetrical, with expression along the ventral midline being brighter and broader at the center (Fig. 2c and c′). This is in contrast to the initiation of *fog* transcription which is reported to be stochastic along the anterior–posterior axis (Lim *et al.* 2017).

At the onset of gastrulation, expression of Fog::sfGFP was clearly visible in cells of the ventral furrow undergoing apical constriction (Fig. 2d and d′, yellow arrowhead); basal Fog::sfGFP could be detected in the stretched cells flanking the furrow (Fig. 2d and d′, pink arrowhead). In the lateral blastoderm, GFP-positive puncta were detected at cell vertices (Fig. 2d, white arrowheads). Interestingly, we consistently observed a few cells in the embryo with upregulated Fog expression (Fig. 2 d and d′, blue arrowhead). Such cells were randomly distributed and did not exhibit any pattern with respect to location. In addition to these cells, we also observed faint Fog::sfGFP expression as 2 distinct stripes along the dorso-ventral (D-V) axis (Fig. 2e)—a pattern that is also observed with *fog* mRNA (Morize *et al.* 1998).

Finally, as with *fog* mRNA, we could detect strong expression of Fog::sfGFP in the posterior midgut primordium (Fig. 2f). At this stage, staining was also clearly visible in the PGCs present atop the primordium (Fig. 2f).

### Fog::sfGFP is expressed in the trachea, amnioserosa, and the epidermis

**Fig. 3.**
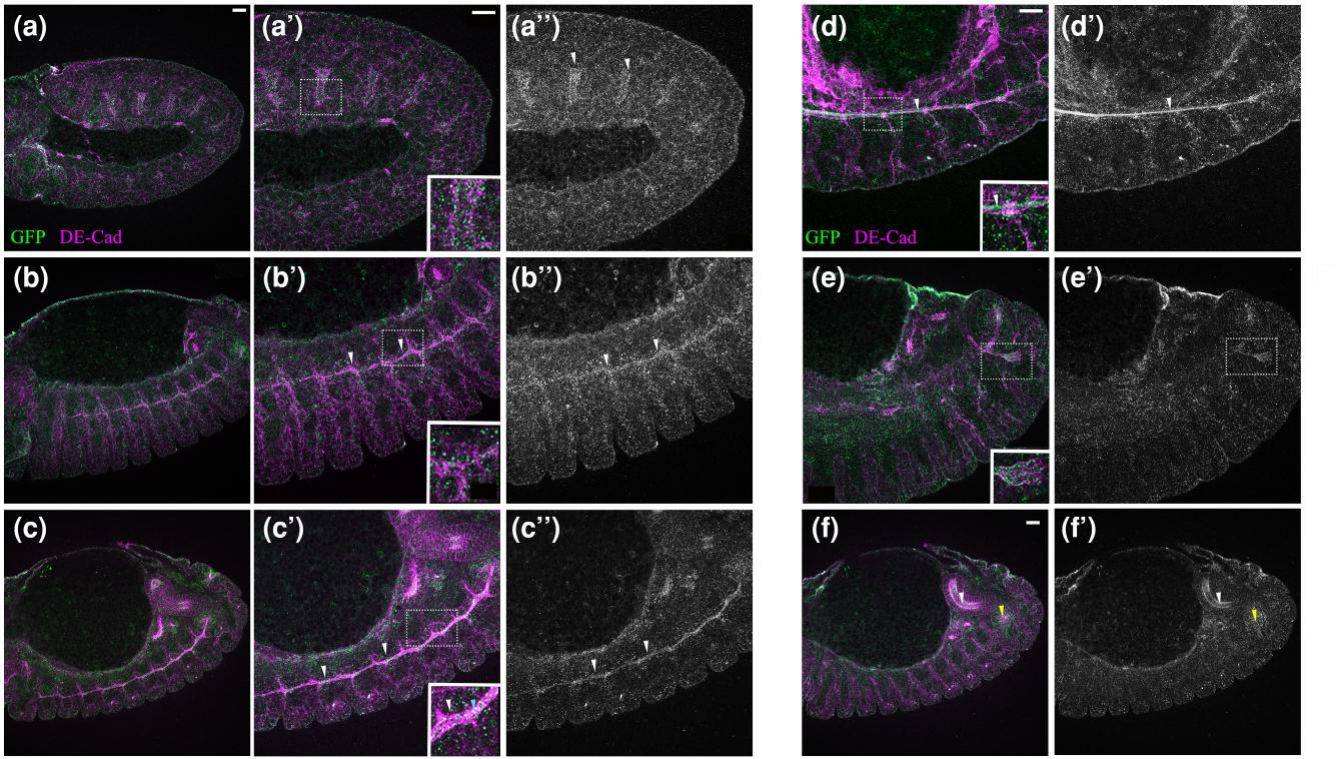
Fog::sfGFP is expressed in developing trachea. Shown are embryos stained with anti-GFP and DE-Cadherin. Gray scale images depict expression of Fog::sfGFP alone. a–a″) Stage 11 embryo. Fog::sfGFP expression in cells surrounding the tracheal pits (see arrowhead in a''). b–b″) Embryonic stage 13. GFP expression is detected along the extending dorsal trunk (arrowheads). c–c″) Stage 14 embryo shows localization of Fog::sfGFP in the lumen of the fused dorsal trunk shown by the blue arrowhead in the inset. Enriched expression observed at junction of 2 adjacent segments is indicated by white arrowheads. d, d′, e, e′) Stage 15 embryo. Localization of Fog::sfGFP in the dorsal trunk is luminal (d, arrow head; inset); punctate expression is seen in secondary branches (e, inset). f, f′) Expression of Fog::sfGFP in the midgut and placode for the posterior spiracle is indicted by white and yellow arrowheads respectively. Scale bar is 20 μm.

To identify other tissues in the embryo that express the ligand, we examined embryos at different developmental stages. It is known that *fog* mRNA is expressed and required during the development of the SG (Lammel and Saumweber 2000; Chung *et al.* 2017). Consistent with this, we observed robust expression of Fog::sfGFP in these cells (Supplementary Fig. 1). Interestingly, we also detected expression of the protein in the developing trachea. At stage 11, GFP signal was detected in cells surrounding the tracheal pits (Fig. 3a–a″); at early stage 13, the signal localized close to DE-Cadherin staining in the developing dorsal trunk (Fig. 3b–b″). At stage 14, however, the protein appeared to be in the lumen of the fused dorsal trunk (Fig. 3c–c″). Expression also appeared elevated at the junction between dorsal trunks of 2 adjacent segments (Fig. 3c–c″). Toward late stage 14, the protein could be detected prominently in the lumen (Fig. 3d and d′). In addition to the dorsal trunk, expression of Fog::sfGFP was also detected in the developing secondary and tertiary branches (Fig. 3e and e′, inset), the midgut, and the placode of the posterior spiracle (Fig. 3f and f′).

**Fig. 4.**
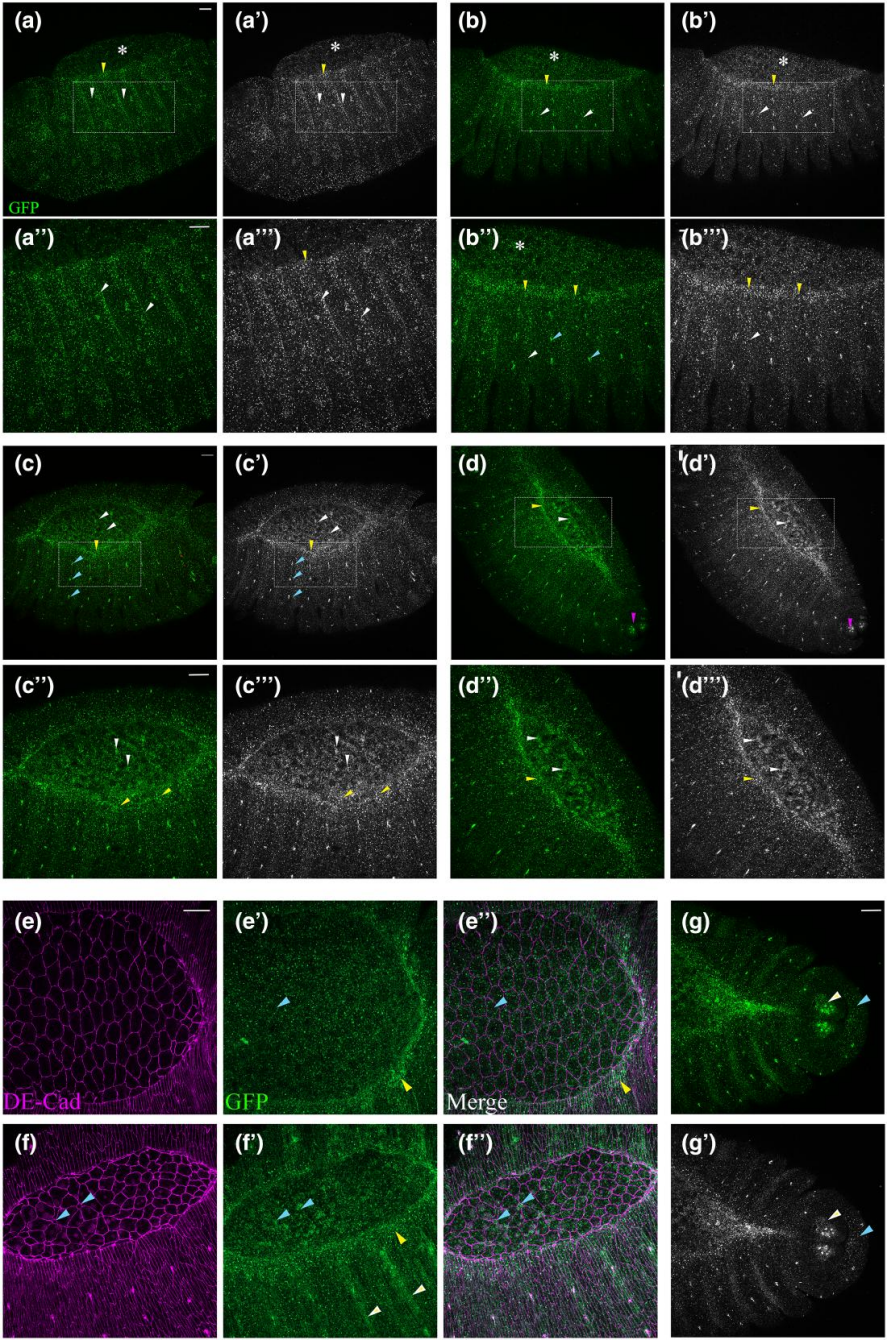
Fog::sfGFP expression in the epidermis and amnioserosa during dorsal closure. a–a‴) Stage 13 embryo at the initiation of dorsal closure. Discrete Fog::sfGFP puncta in the epidermis, and, leading edge cells, are indicated by white and yellow arrowheads respectively; expression in the amnioserosa is indicated by an asterisk. b–b‴) Stage 14 embryo shows more prominent expression of Fog::sfGFP in the leading edge cells. c, d) Stage 15 and late 15 embryo respectively, undergoing dorsal closure. Strong GFP staining is seen at the leading edge and in amnioserosa. Blue arrowheads indicate expression associated with peripheral sensory neurons. Magenta arrowheads mark cells in the placode for posterior spiracles. e–f) Embryo at early (stage 14, e) and late stages of dorsal closure (stage 15, f) stained with GFP and DE-Cadherin. An increase in GFP signal is seen in cells undergoing apical constriction (f, blue arrowheads). g, g′) Fog::sfGFP expression in the placode for posterior spiracles and anal pad indicated by white and blue arrowheads respectively. Scale bar for all is 20μm.

Through stages 13–15, we also observed dynamic expression of the protein in the lateral epidermis and amnioserosa (Fig. 4). At stage 13, punctate GFP signal was detected in each segment along the D-V (Fig. 4a and a′, white arrowheads) axis and at the leading edge of epidermal cells bordering the amnioserosa (Fig. 4a and a′, yellow arrowhead). At this stage, expression in the amnioserosa though clear did not appear prominent (Fig. 4a and a′). However, through dorsal closure, Fog::sfGFP signal at the epidermal leading edge and amnioserosa increased progressively (Fig. 4b–d). Upon costaining with DE-Cadherin, we observed that at stage 14, most of the GFP signal in amnioserosa is present laterally along the cell margin (Fig. 4e–f). However, during dorsal closure (stage 15), expression of Fog::sfGFP is upregulated with the signal being more intense in cells undergoing apical constriction (Fig. 4f). At this stage, we could also detect GFP signal in the placode of the posterior spiracle and in the anal pad (Fig. 4g).

### Fog::sfGFP is prominently expressed in glia

**Fig. 5.**
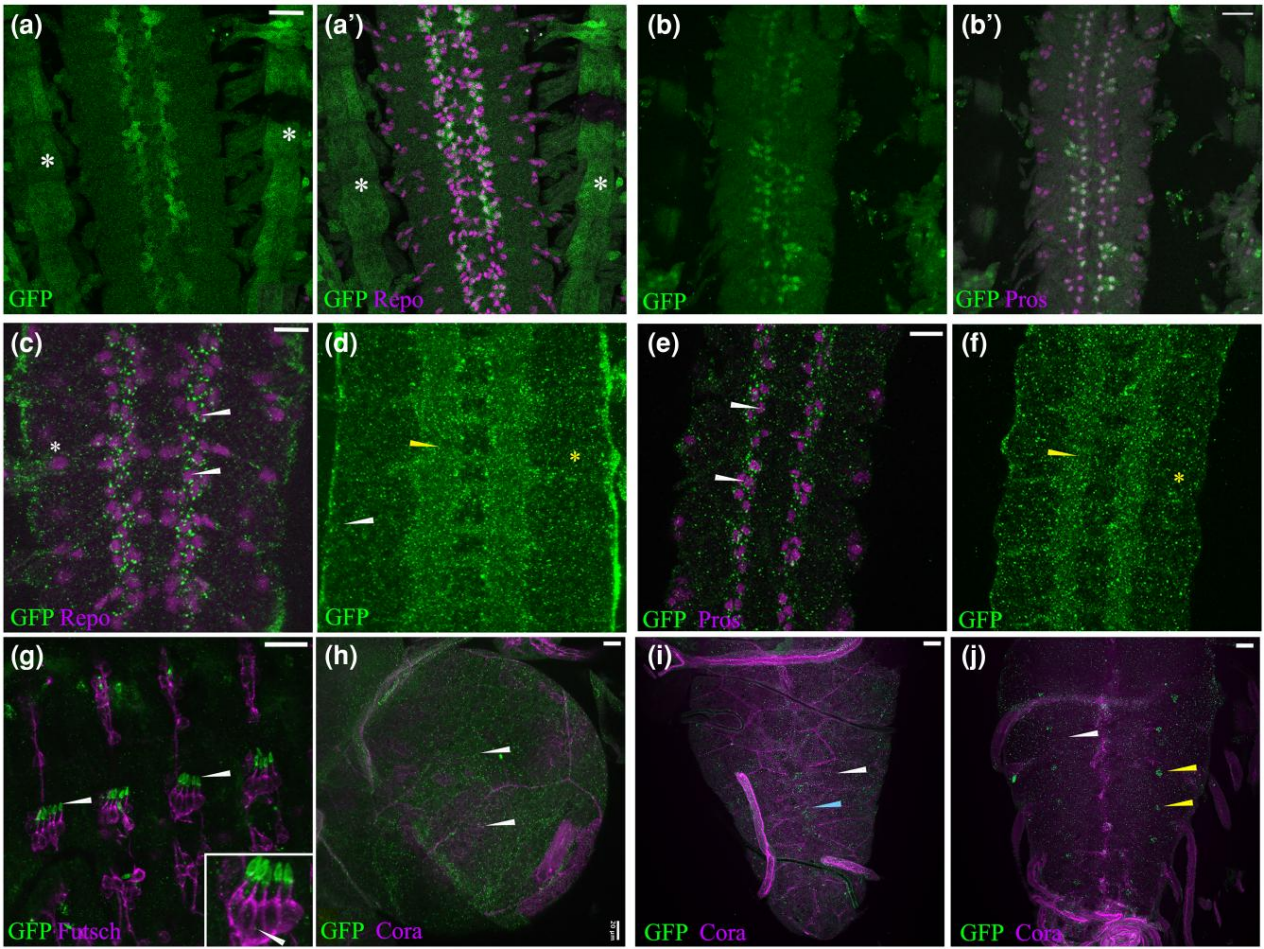
Expression of Fog::sfGFP in the nervous system. a, b) Embryonic CNS of a *fog*^MiMiC^ embryo stained with anti-Repo and anti-GFP (a and a'); anti-Pros and anti-GFP (b, and b′). Note the overlap in expression of GFP and Pros. Expression is also seen in ventrolateral muscles (a and a′, asterisk). c–f) Fog::sfGFP expression in the embryonic CNS. Enriched GFP staining is seen in LG marked by Repo (c, arrowheads) and Pros (e, arrowheads); expression in ML-SPG is marked by asterisk in c). d, f) Fog::sfGFP expression in the neuropil and underlying neuronal region is marked by yellow arrowhead and asterisk respectively. g) Fog::sfGFP expression (arrowheads) associated with sensory neurons in the embryonic PNS. h–j) Third instar larval brain and VG. Fog::sfGFP expression indicated by white arrowheads is seen in SPGs outlined by anti-coracle. In addition, midline expression indicated by blue arrowhead is seen on the ventral side of the ganglion (i); expression in specific lateral cells is seen on the dorsal side (j, yellow arrowhead). Scale bar for a–b′) and e–j) is 20 μm; for c) and d), scale bar is 10 μm.

We have previously reported the expression of *fog* mRNA in the embryonic CNS and peripheral nervous system (PNS). In the CNS, expression of *fog* is enriched in a subset of dorsally positioned longitudinal glia (LGs) and expression in these cells is stochastic with no two embryos having the same pattern of expression in LG (Ratnaparkhi and Zinn 2007). Interestingly, we observed this stochasticity in the *fog* MiMIC line (*fog^MiMiC^*) that carries the GFP-containing MiMIC cassette within the approximately 10-kb intron present directly 5′ to the coding exon (Fig. 5a and a′).

LGs are known to arise from a common precursor glioblast (Schmidt *et al.* 1997), and a subset of these that express transcription factor Pros differentiate into larval astrocytes (Stork *et al.* 2014). We checked if LGs that express the *fog* MiMIC reporter (GFP) belong to this specific subclass of glia and found that all GFP-expressing LGs are positive for Pros (Fig. 5b and b′) indicating that *fog* is indeed expressed in astrocyte precursors and is likely to have roles in astrocyte development.

The expression pattern of Fog::sfGFP in the embryonic CNS matched *fog* mRNA in that the most prominent GFP staining was associated with LG (Fig. 5c). Further, as observed with the MiMIC line, this expression was also associated with Pros-positive LG (Fig. 5e). However, in the absence of a membrane marker to delineate cell boundaries, it was difficult to determine whether expression was restricted to Pros-positive LG alone.

In addition to LG, we detected Fog::sfGFP expression around other Repo-positive nuclei including those located mediolaterally (Fig. 5c), which we predict, based on position and large nuclear size, to be subperineurial glia (SPGs) that give rise to the blood–brain barrier (BBB).

In more ventral sections, expression was detected throughout the CNS including the neuropil which is the synaptic region (Fig. 5d–f), indicating widespread Fog::sfGFP expression in the underlying neurons and possibly other glia as well. In the periphery, consistent with the expression pattern of *fog* mRNA (Ratnaparkhi and Zinn 2007), we detected strong GFP expression in scolopale cells that ensheath the dendrite of sensory neurons (Figure 5g) with faint expression in the neurons (Fig. 5g, inset).

Toward the end of embryogenesis, SPGs expand and establish pleated septate junctions with their neighbors to ensheath the entire brain and form the BBB. To confirm Fog expression in SPGs, we checked for expression of the protein in the 3rd instar larval brain and found Fog::sfGFP puncta distributed in the SPGs of both optic lobes (Fig. 5h) and the ventral ganglion (VG; Fig. 5i and j). On the dorsal side of the VG, enriched expression was observed in specific cells laterally positioned on either side of the neuropil (Fig. 5j,).

In summary, these data indicate that during development, Fog signaling is likely to play an important role in the nervous system, particularly glia. Its expression in the SPGs suggests a role for this signaling pathway in the formation of the BBB.

### Identification of *cis*-regulatory elements regulating *fog* expression in the CNS

The above results reveal a complex spatiotemporal expression pattern for *fog* during embryonic development. In the CNS itself, *fog* is expressed in neurons and multiple types of glia. Furthermore, in the LG, where *fog* expression is enriched, regulation appears to be stochastic. As a step toward understanding the underlying mechanisms that regulate expression, we sought to identify *cis*-regulatory sequences that control spatiotemporal expression of the gene. To this end, we generated individual reporter lines using overlapping intronic sequences from the single approximately 10-kb intron in the *fog* gene and examined reporter expression (GFP or RFP) in the CNS. Here, we present an analysis of the expression pattern of 3 such enhancer–reporter lines spanning a region of approximately 3.5-kb upstream of the translation start site. Cloning of the more distal intronic sequences proved difficult due to the presence of long stretches of nucleotide repeats.

**Fig. 6.**
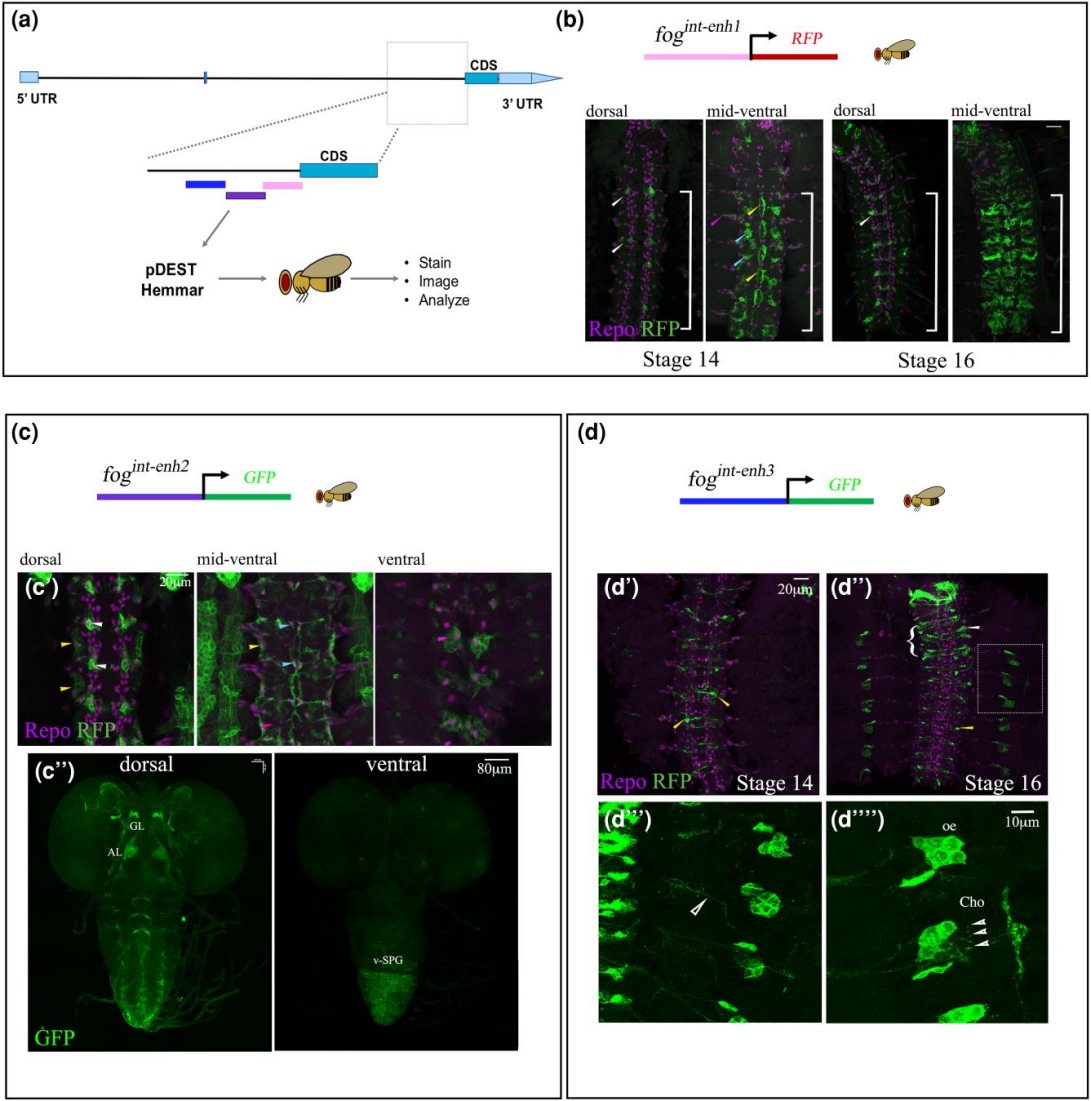
Intronic enhancers drive expression in distinct subsets of glia and neurons in the embryonic CNS. a) Schematic representation of the intronic regions used to generate reporter lines. b) Diagrammatic representation of *fog^int-enh1^::RFP.* Expression pattern of this reporter in the CNS of a stage 14 and stage 16 embryo stained with anti-Repo and anti-RFP. The bracket denotes abdominal segments. Reporter expression is seen in specific dorsally placed LG (white arrowhead); in the mid-ventral region, expression is seen in CBG (blue and yellow arrowheads) of the abdominal segments. c) Diagrammatic representation of *fog^int-enh2^::GFP*. c′) In the CNS, expression is seen in multiple subtypes of glia including LG (white arrowhead), lateral SPG (yellow arrowhead), medial CBG (light blue arrowhead), and ventral SPG (pink arrowhead. c″) In the larval brain, expression is seen in the developing AL, axons in the brain and VNC, and ventral SPG in VNC. d) Diagrammatic representation of *fog^int-enh3^::GFP* d′–d″) Bracket denotes thoracic segments. Expression pattern of *fog^int-enh3^::GFP* in thoracic and abdominal interneurons (d′ and d″, white and yellow arrowheads, respectively), motor axons (d‴, hollow arrowhead), oenocytes (d‴, oe), and chordotonal organs (d‴, Cho). Scale bar in all panels is 20 μm; in c″, scale bar is 80 μm.

**Fig. 7.**
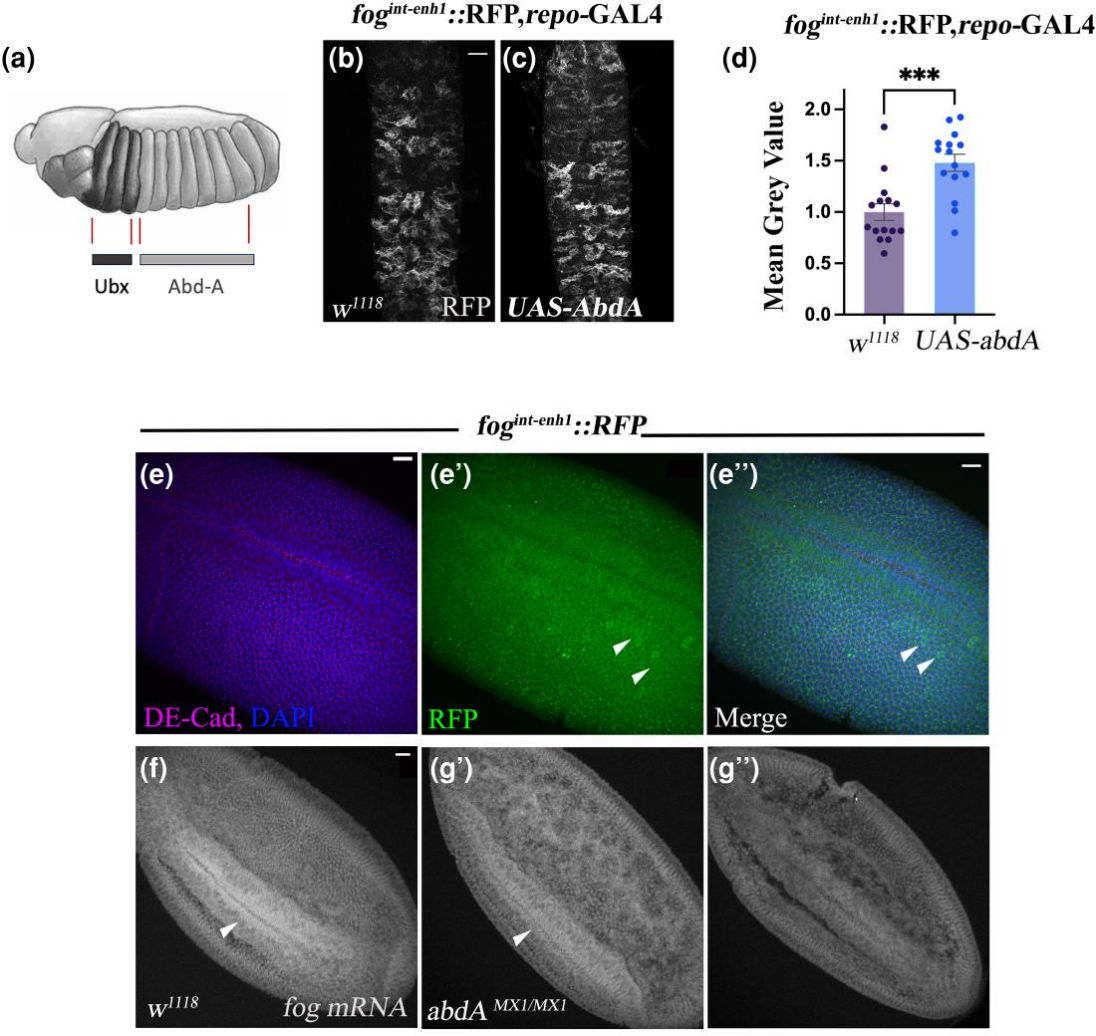
*fog* ^*int-enh1*^ is regulated by Hox protein *AbdA*. a) A schematic showing the expression pattern of AbdA in embryos. b, c) *fog^int-enh1^::RFP, repo-GAL4/+* b) and *fog^int-enh1^::RFP, repo-*GAL4 *> UAS-abdA* c) stained with anti-RFP. Note increase in reporter expression in c). d) Quantification of reporter expression. Overexpression of AbdA leads to approximately 30% increase in staining intensity of the reporter (control mean gray value: 1 ± 0.05; *UAS-abdA*: 1.3 ± 0.07). e–e″) *fog^int-enh1^::RFP* embryo at gastrulation stained with anti-DECad, anti-RFP, and DAPI (blue). Note expression of RFP in the ventral furrow and lateral blastoderm. Strong reporter expression is observed a few scattered cells in the lateral blastoderm (white arrowheads). f, g) *fog* mRNA expression in wild-type (f) and homozygous *abdA^MX1^* mutant (g, g′). Transcript levels are reduced in the mutants. Scale bar in (a) and (b) is 20 μm; scale bar (e–g′) is 40 μm; all values in the graph are represented as mean ± SEM; Student's *t*-test was used for comparison; * indicates *P* < 0.001.

Interestingly, all 3 reporters showed expression in the CNS albeit in different cell types. It is to be noted that the expression of these enhancers is not exclusive to the CNS. Intronic enhancer-1 (hereafter referred to as *fog^int-enh1^*), located immediately upstream to the translation start site (Fig. 6a), also drives expression in the ventral furrow (Fig. 7e); expression of intronic enhancer-2 (*fog^int-enh2^*) is seen in the SGs, fat body (data not shown), and somatic musculature; activity of *fog^int-enh3^* is detected in oenocytes. In the CNS, expression of *fog^int-enh1^* and *fog^int-enh2^* was primarily in glia while *fog^int-enh3^* expression was in neurons.

Glia are of different types and can be identified on the basis of their morphology and position in the VNC. For instance, SPGs in the embryo grow into large polygonal cells that ensheath the brain. These cells are polyploid and therefore can be recognized on the basis of their position and nuclear size. The different types of glia and their positions have been described in detail by Beckervordersandforth *et al.* (2008). We used this study as an atlas to identify glia that express the different reporters.

*fog^int-enh1^*::RFP drives expression in glia. In the dorsal region, we found expression of the reporter to be restricted to a single mediolaterally placed LG; ventrally, expression was specific to medial and ventral CBG (Fig. 6b). Interestingly expression in these CBG was restricted only to abdominal segments with occasional faint expression in thoracic segments. The near absence of staining in thoracic CBG was particularly striking in early embryos although at late stage 16, faint reporter expression could be discerned in the thoracic segments as well (Fig. 6b). In addition to the CBGs, expression of this enhancer was also detected in peripheral glia (Fig. 6b).

Unlike *fog^int-enh1^*, the expression pattern of intronic enhancer-2 (hereafter referred to *fog^int-enh2^*) was more broad: In the dorsal regions, robust expression was seen in 1–2 laterally placed LG per hemisegment whose pattern seemed similar to that of the *fog^int-enh1^::RFP* line (Fig. 6c). In this plane, reporter expression was also seen in dorso-lateral SPG located along the lateral edges of the CNS (Fig. 6c). In the mid-ventral region, we detected reporter expression in lateral SPG, Mediolateral SPG (ML-SPG; Fig. 6c), and medial CBG (Fig. 6c). Finally, in the ventral region, this enhancer was found to express in the medio-ventral SPG (Fig. 6c).

Interestingly, in the third instar larval brain, this enhancer was seen to drive expression in both neurons and glia: On the dorsal side, expression was seen in the antennal lobe (AL), the likely gamma lobe of the mushroom body, and the VNC (Fig. 6c). However, on the ventral side, expression was specific to ventral SPGs of the abdominal segments in the VNC. In summary, the expression pattern of this enhancer appears to be primarily glial during embryogenesis but switches to driving expression in both glia and neurons during the larval stages.

In contrast to enhancer-1 and -2, expression of enhancer-3 (*fog^int-enh3^::GFP)* was neuronal with expression restricted to a couple of neurons per segment. Like *fog^int-enh1^::RFP*, we observed a difference in expression pattern between the abdominal and thoracic segments. In the former, staining was restricted to a pair of contralaterally projecting interneurons; in the latter, 2 pairs of interneurons were seen expressing the reporter (Fig. 6d′ and d″). In the periphery, we could detect faint expression in motor axons, which is consistent with the reported role of Fog signaling in motor axon guidance (Ratnaparkhi and Zinn 2007). This expression appeared more distinct in late stage 16 embryos (Fig. 6d″).

In the dorsal epidermis, reporter expression was seen in a cluster of 6 cells in each hemisegment. Based on morphology and position, we believe these cells to be oenocytes that are associated with lipid storage (Fig. 6d‴). Ventral to the oenocytes, reporter expression could be detected at the tips of sensory neurons in the chordotonal organs (CHOs; Fig. 6d‴). Neurons of the CHO and oenocytes are located close to each other. Indeed, signals from the developing dorsal CHO regulate the differentiation of oenocytes (Elstob *et al.* 2001).

### Activity of *fog^int-enh1^* is regulated by AbdA

In the embryonic CNS, expression of AbdA, the homeobox-containing transcription factor, is restricted to the abdominal segments A1–A7 (parasegments 7–12; Hirth *et al.* 1998). Here, AbdA plays a crucial role in establishing segmental identity and patterning. Because expression of *fog^int-enh1^* is restricted to the abdominal segments, we sought to test whether this enhancer is responsive to AbdA. As a first step, we carried out an in silico analysis using JASPAR to identify potential AbdA-binding sites in the *fog^int-enh1^* sequence. At a threshold of 0.85, we identified a single AbdA-binding motif in the 1.1-kb sequence of *fog^int-enh1^* (Supplementary Fig. 2a and b). Multiple motifs were identified at a lower threshold of 0.8. We also identified binding motifs for Hox protein Ultrabithorax (Ubx) and cofactor Homothorax. Interestingly, both AbdA and Ubx were found to share the same binding motif with different matrix scores (Supplementary Fig. 2c).

Next, we overexpressed AbdA in the background of *fog^int-enh1^*::RFP using the pan-glial *repo*-GAL4 that expresses in differentiated glia and checked for a change in reporter expression using immunofluorescence. Compared with control (Fig. 7b), we found the intensity of RFP staining to be nearly 30% higher in embryos overexpressing AbdA (Fig. 7c and d). In addition, these embryos also showed increased RFP expression in the thoracic segments indicating upregulated enhancer activity in these segments.

As stated in the previous section, *fog^int-enh1^*::RFP expresses in early blastoderm embryos. Through immunostaining, we detected expression of RFP in both the blastoderm and the presumptive mesoderm at the ventral furrow (Fig. 7e–e″). Furthermore, similar to Fog::sfGFP embryos that show a few scattered cells with enriched expression of Fog::sfGFP (Fig. 1), we observed enriched RFP expression in randomly positioned cells in the blastoderm (Fig. 7e′ and e″, arrowheads). This observation, together with the results from *abdA* overexpression, prompted us to check whether the expression of *fog* mRNA in the ventral furrow is downregulated in *abdA* mutants. We performed RNA in situs against *fog* using a rhodamine-labeled anti-DIG antibody to detect the DIG-labeled RNA probes. Indeed, compared with control (Fig. 7f), expression of *fog* mRNA appeared considerably reduced in *abda^mx1/mx1^* embryos (Fig. 7g and g′) with some embryos showing greater reduction in signal than others. Given this decrease in staining, we checked if *abda* mutant embryos exhibit furrow defects. For this, we performed the experiment using non-fluorescent color-based reaction to detect the probe. Despite the reduction in signal, about 60% of the mutants seemed to have relatively normal-looking furrow while the remaining 40%, showed furrow defects suggestive of a delay in invagination (Supplementary Fig. 3). Together, these results suggest a role for AbdA in regulating *fog* expression.

## Discussion

We have carried out a detailed characterization of Fog expression during embryogenesis and the larval CNS using a genome-engineered Fog::sfGFP line generated using CRISPR technology. The reasons to believe that this reagent faithfully represents Fog expression are the following: First, we could detect the expression of Fog::sfGFP in all the tissues previously known to express Fog. Second, when we mined the embryo single-cell RNA-seq data available at https://www.ebi.ac.uk/gxa/home (Karaiskos *et al.* 2017), we found widespread expression of *fog* in almost all clusters (Supplementary Fig. 4a). In cluster 2 that expresses polar granule component (pgc, Martinho *et al.* 2004), a gene that is strongly expressed in PGCs, we found a significant overlap between *pgc* and *fog* (Supplementary Fig. 4a). Although not all *pgc* expressing cells are likely to be pole cells/PGCs, the overlap in expression between *pgc* and *fog* supports our observation on Fog::sfGFP expression in pole cells.

We also mined the single-cell RNA-seq data of larval brains (https://cells.ucsc.edu; Brunet Avalos *et al.* 2019). Here too, we found *fog* expression distributed across multiple cell clusters. This data set identified 2 glial clusters (Supplementary Fig. 4b, magenta cluster) and one of them showed a strong overlap between *repo* and *fog* (Supplementary Fig. 4b). Together, these data support our observations on Fog expression in the nervous system.

Finally, when we downregulated *fog* expression in embryos using *repo*-GAL4, we observed a distinct decrease in GFP staining (Supplementary Fig. 5) which indicates that the GFP signal we have observed is indeed that of Fog. A caveat that would be useful to note is that because Fog is an extracellular ligand, it is possible that the site at which it is detected may not be the site at which it is made. These details though currently beyond the scope of this study will need to be resolved based on context.

Our analysis of Fog::sfGFP has revealed aspects of Fog expression, localization, and regulation that have hitherto not been described previously. We find that Fog::sfGFP is expressed very early during embryogenesis, at the very onset of cellularization. Punctate expression is seen around nuclei at this stage that becomes more prominent along the lateral membrane as cellularization progresses. The presence of Fog::sfGFP at these stages suggests that it is derived from the maternal component. While it is known that *fog* mRNA is maternally deposited in the embryo (Costa *et al.* 1994), its localization and function have not been explored in detail. Localization of Fog along the lateral membrane during cellularization suggests a possible role for maternal Fog in regulating myosin activity for extension of the lateral membrane during cellularization. Similarly, Fog expression in the PGCs is also likely to be maternal although this will need to be confirmed. Expression of Fog in germ cells has not been reported previously. It would be interesting to test whether Fog signaling plays a role in maintaining PGCs together as a cluster in the PMG analge during germ-band extension.

In the embryo we detect expression of Fog in multiple tissues that undergo extensive cell and tissue morphogenesis including the SGs (Supplementary Fig. 1), trachea, amnioserosa, and the extending lateral epidermis during dorsal closure. The trachea and SGs represent tubular organs. While the role of Fog signaling in the development of the SG is well documented (Chung *et al.* 2017; Le and Chung 2021; Vishwakarma *et al.* 2022), there is still much to be understood about how this pathway regulates tube formation in the trachea. It would be interesting to determine if distinct signaling mechanisms, using tissue-specific receptors, operate in each of these contexts. To date, GPCRs Mist and Smog are the 2 known Fog receptors that function during gastrulation. Smog also plays a role during SG morphogenesis (Vishwakarma *et al.* 2022). Interestingly, neither receptor mediates Fog signaling in the embryonic CNS. Rather, Smog functions as a negative regulator of Fog signaling in this context (Shweta *et al.* 2021). This suggests that there are likely to be multiple receptors for Fog and these remain to be identified.

Of particular interest is the expression of Fog::sfGFP in the amnioserosa and lateral epidermis during dorsal closure. It is well documented that delamination of cells in the amnioserosa involves apical constriction and is regulated by DRhoGEF2 (David *et al.* 2010; Gorfinkiel and Blanchard 2011; Azevedo *et al.* 2011). Furthermore, this process is also regulated by mitochondrial fusion and fission (Muliyil *et al.* 2011). In an earlier study, we have shown that mitochondrial fission/fragmentation is an event downstream of Fog signaling and inhibiting mitochondrial fission attenuates Fog signaling (Ratnaparkhi 2013). It would be of interest to test whether Fog signaling in the amnioserosa regulates mitochondrial fragmentation essential for apical constriction.

Dorsal closure also involves the extension of the lateral epidermis over the amnioserosa followed by zippering, which requires coherence at the leading edge and tight cell–cell coupling (Jacinto *et al.* 2002). This process involves extensive cell stretching, powered by the accumulation of nonmuscle myosin II at the apical end of cells at the leading edge (Kiehart *et al.* 2000; Kiehart *et al.* 2017). Our observations show that as zippering progresses expression of Fog::sfGFP in cells of the leading edge (Fig. 4d). The process of zippering is powered by actomyosin contractility. It would be of interest to determine whether Fog signaling is involved in regulating the accumulation of nonmuscle myosin essential for driving this process.

### Fog expression in the CNS

Our work has previously shown that Fog signaling in neurons regulates motor axon guidance; in LGs that show enriched expression of *fog mRNA*, loss of Fog signaling leads to defects in glial organization and morphogenesis. (Ratnaparkhi and Zinn 2007). Our analysis of the expression pattern of Fog in the CNS not only supports our previous findings but also predicts a wider role for this signaling pathway in the development of the nervous system. Further, the fact that Fog::sfGFP is expressed in multiple subtypes of glia including astrocyte precursors suggests a prominent role for Fog signaling in glial development and/or morphogenesis with the mechanism of signaling and its regulation likely being unique in each case.

It is to be noted that activation of FGFR/Heartless signaling in astrocytes regulates the extension and growth of glial processes (Stork *et al.* 2014). Our previous study has shown that FGFR/Heartless signaling negatively regulates the Fog pathway in the LG (Shweta *et al.* 2021), a subset of which give rise to astrocytes. It would be interesting to determine whether Fog signaling regulates astrocyte development and/or morphogenesis and whether this is through the regulation of FGFR/Heartless signaling.

That Fog is also expressed in neurons brings in another layer of complexity to the working of this pathway (Figs. 5 and 6): How does a neuron or glia distinguish between an autocrine signal emanating from itself and an exocrine signal coming from the neighboring glia or neuron? Identifying mechanisms that help compartmentalize and regulate signaling in each of these cellular contexts would help shed light on how the Fog signal is differentially perceived.

### Role of Hox genes in regulating *fog* expression

In this study, we describe the expression pattern of 3 intronic enhancers namely *fog^int-enh1^*, *fog^int-enh2^*, and *fog^int-enh3^*. An interesting finding to emerge from these analyses is the possible role of *hox* genes in regulating *fog* expression. Using gain-of-function analyses, we demonstrate that the activity of *fog^int-enh1^* is responsive to AbdA (Fig. 7c and d) suggesting a potential role for AbdA in regulating *fog* expression. Supporting this, we find reduced expression of *fog* mRNA in the ventral furrow of *abda* mutants (Fig. 7f, g′, and g″). Additional support is also provided by chromatin immunoprecipitation studies that have identified *fog* as a target of AbdA (Beh *et al.* 2016; Zouaz *et al.* 2017).

It is worth noting that the expression pattern of *fog^int-enh3^::GFP* is complementary to *fog^int-enh1^::RFP* (Fig. 6b and d) with a distinct expression pattern in thoracic segments. Given that hox gene *ubx* is involved in patterning the thoracic segments, it would be interesting to determine the role of this gene in regulating *fog* expression.

Embryogenesis is a dynamic process that involves movement, organization and reorganization of cells. Our results show that Fog is expressed in a wide range of cells and tissues, all of which are dynamic and undergo dramatic morphogenetic changes. With actin remodeling being the key downstream event of morphogenesis, it would appear that Fog regulates actin dynamics in each of these contexts. The nature of regulation in each case, however, is likely to be unique. Exploring these regulatory paradigms that control cell and tissue dynamics to eventually give rise to specificity in shape would be the focus of future studies.

## Supplementary Material

jkae032_Supplementary_Data

## Data Availability

The authors affirm that all data necessary for the conclusions drawn in this article are represented within the article. Plasmids and flystocks generated for this study are available on request. Web sources are as follows: FlyBase: https://flybase.org (Gramates *et al.* 2022); UCSC Cell Browser: https://cells.ucsc.edu (Speir *et al.* 2021); single-cell expression atlas (EMBL-EBI) database: https://www.ebi.ac.uk/gxa/home (Brunet Avalos *et al.* 2019); JASPAR: http://jaspar.genereg.net/ (Castro-Mondragon *et al.* 2022); and MEME suite database: https://meme-suite.org/meme/tools/tomtom (Bailey *et al.* 2015). Supplemental material available at G3 online.
