# Supplementary material for: Embryonic spatiotemporal expression pattern of Folded gastrulation suggests roles in multiple morphogenetic events and regulation by AbdA
Source: G3 (Bethesda). 2024 Feb 15;14(5):jkae032. doi: 10.1093/g3journal/jkae032 (PMC11653764; doi:10.1093/g3journal/jkae032)
Supplement: jkae032_Supplementary_Data [file jkae032_supplementary_data.zip › Supplementary Material-Figure Legends-Proofed.docx]

**Supplementary Material**

**Figure Legends:**

Figure S1: **Expression of Fog::sfGFP in the salivary glands**.

(A-B) Color and grey scale images of Fog::sfGFP expression in salivary glands (arrow). Staining in the cells appears punctate. Expression is also seen in the lumen.

Figure S2: **Sequence analysis of *fog^int-enh1^***. (A-B) Sequence of *fog^int-enh1^*. Highlighted are binding sites for Hox proteins AbdA, Ubx and Hth at a threshold of 0.85. Note presence of common motif for AbdA and Ubx. (B-C) Scores of the identified Hox motifs (B) and the degree of conservation using position weighted matrix is shown (C).

Figure S3. **RNA *in situ* hybridization for *fog* on wildtype and *abda^MX1/MX1^* mutants**. Compared with control (A), *abda* mutants show reduced *fog* expression (asterisk) and ventral furrow defects (B,C, arrow). Scale bar is 50μm.

Figure S4: **Analysis of single cell RNA sequencing data indicates *fog* expression in multiple cell types in the embryo and larval CNS**.

(A) Embryo Single cell RNA seq data mined from https://www.ebi.ac.uk/gxa/home (Karaiskos et al., 2017) Expression of *fog* is detected in multiple cell clusters. The boxed region marks cluster 2. Expression of *polar granule component* (*pgc*) and *fog* in cluster 2. Note the overlap between *pgc* and *fog* expression.

(B) Larval brain single cell RNA seq data mined from larval *https://cells.ucsc.edu* (Brunet et al., 2019). Expression of *fog* is detected in multiple clusters. The boxed region is the glial cluster (in magenta). Note the overlap between *repo* and *fog* in the glial cluster.

Figure S5: **Validation of the Fog::sfGFP line using RNAi**. Color and grey scale images of the embryonic CNS of Fog::sfGFP/ Fog::sfGFP; *repo*-GAL4/+ (A, A’) and Fog::sfGFP/Fog::sfGFP; *repo*-GAL4>*UAS-fogRNAi* stained for GFP and Repo (B,B’). A decrease in GFP staining is observed in embryos expressing *fogRNAi* (B-B’).
