# Supplementary figures and images for: Embryonic spatiotemporal expression pattern of Folded gastrulation suggests roles in multiple morphogenetic events and regulation by AbdA
Source: G3 (Bethesda). 2024 Feb 15;14(5):jkae032. doi: 10.1093/g3journal/jkae032 (PMC11653764; doi:10.1093/g3journal/jkae032)

Fig. S1

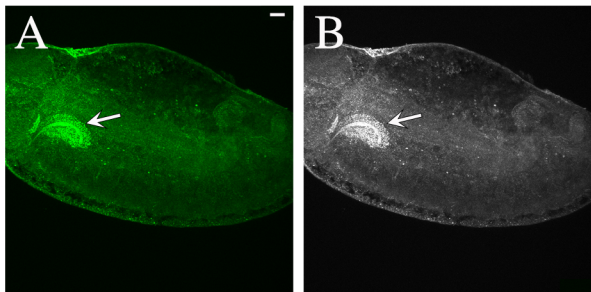

Supplement: jkae032_Supplementary_Data [file jkae032_supplementary_data.zip › Figure_S1_G3-2024-404877.pdf]

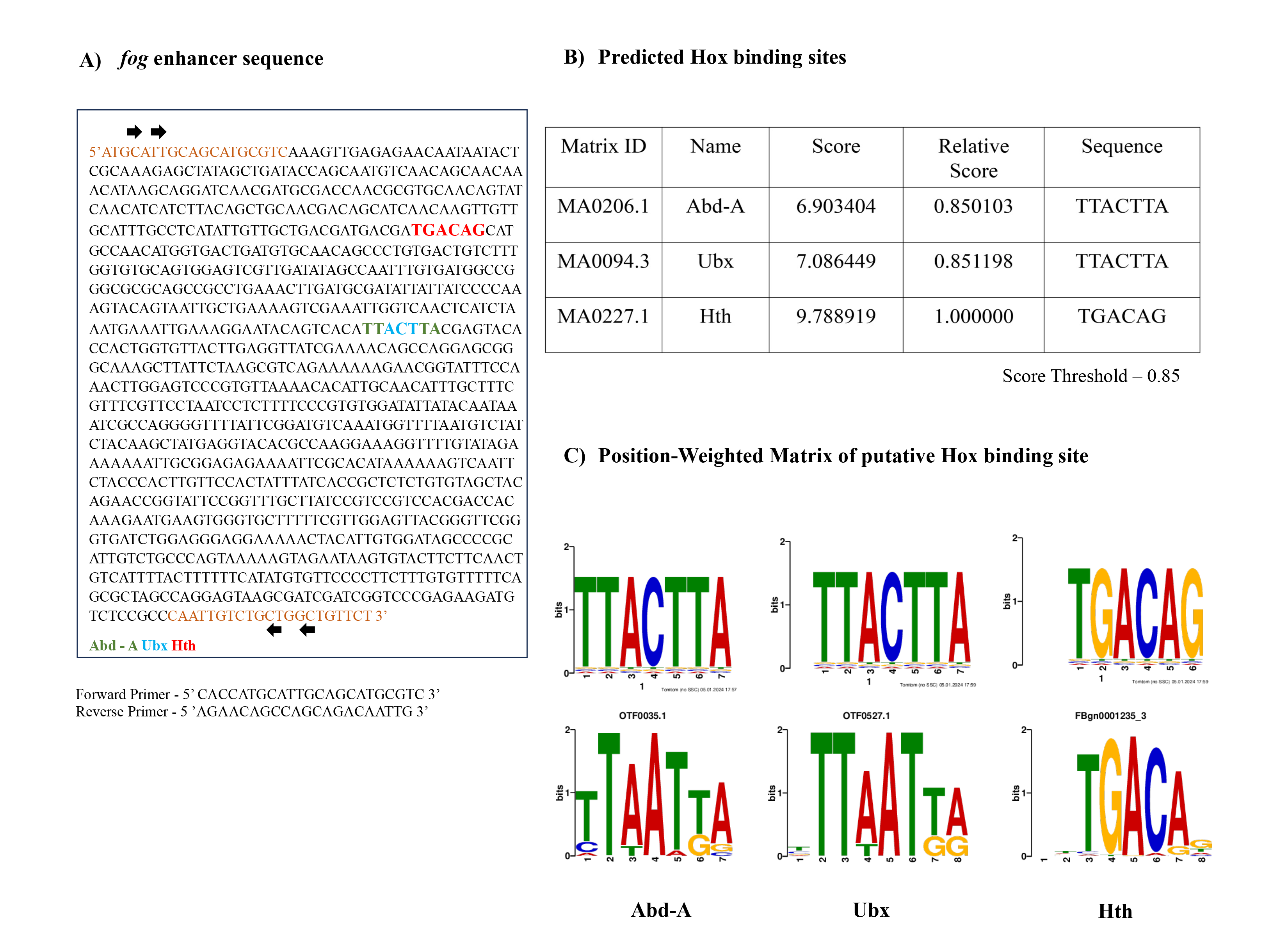

Supplement: jkae032_Supplementary_Data [file jkae032_supplementary_data.zip › Figure_S2_G3-2024-404877.tif]

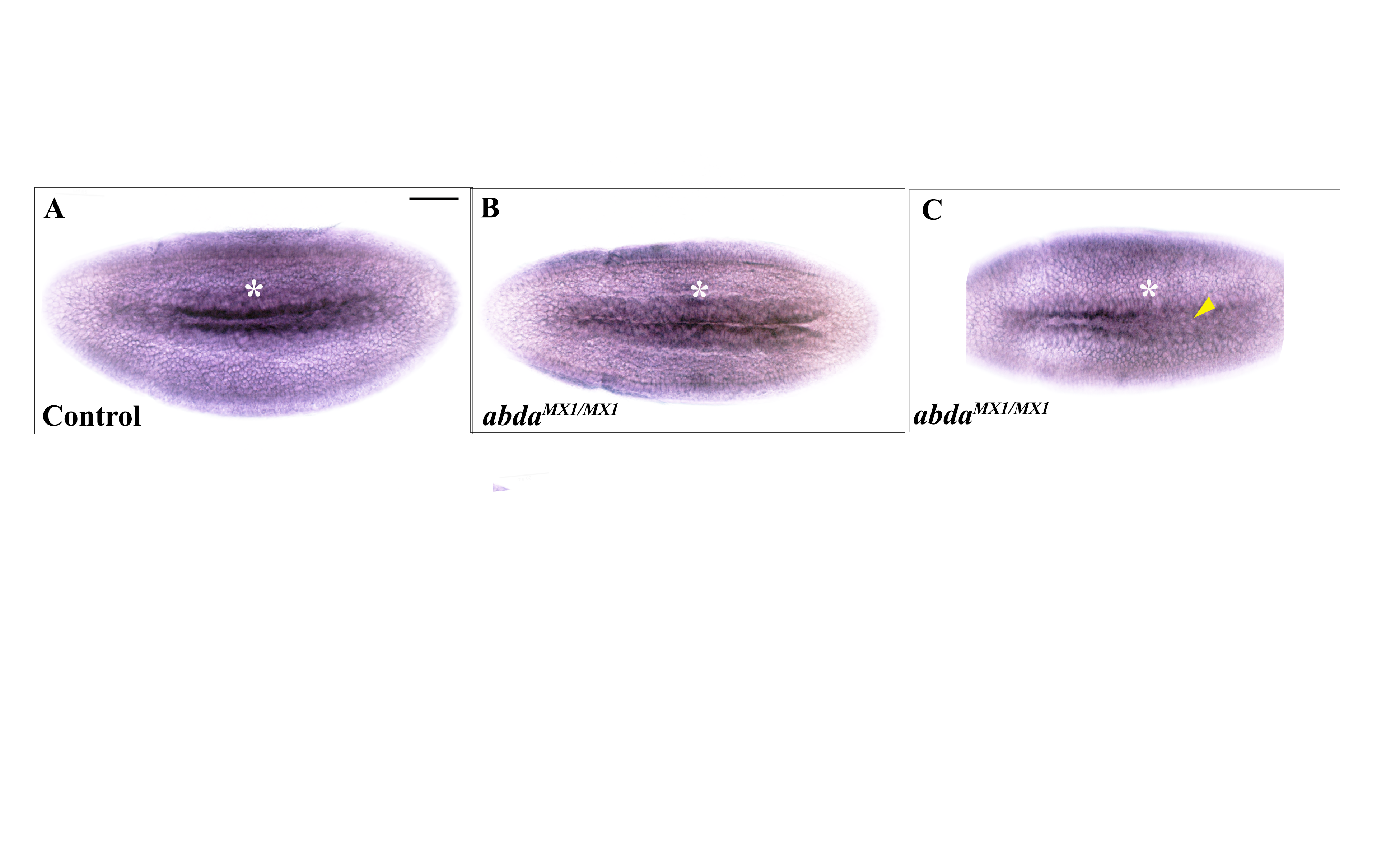

Supplement: jkae032_Supplementary_Data [file jkae032_supplementary_data.zip › Figure_S3_G3-2024-404877.tif]

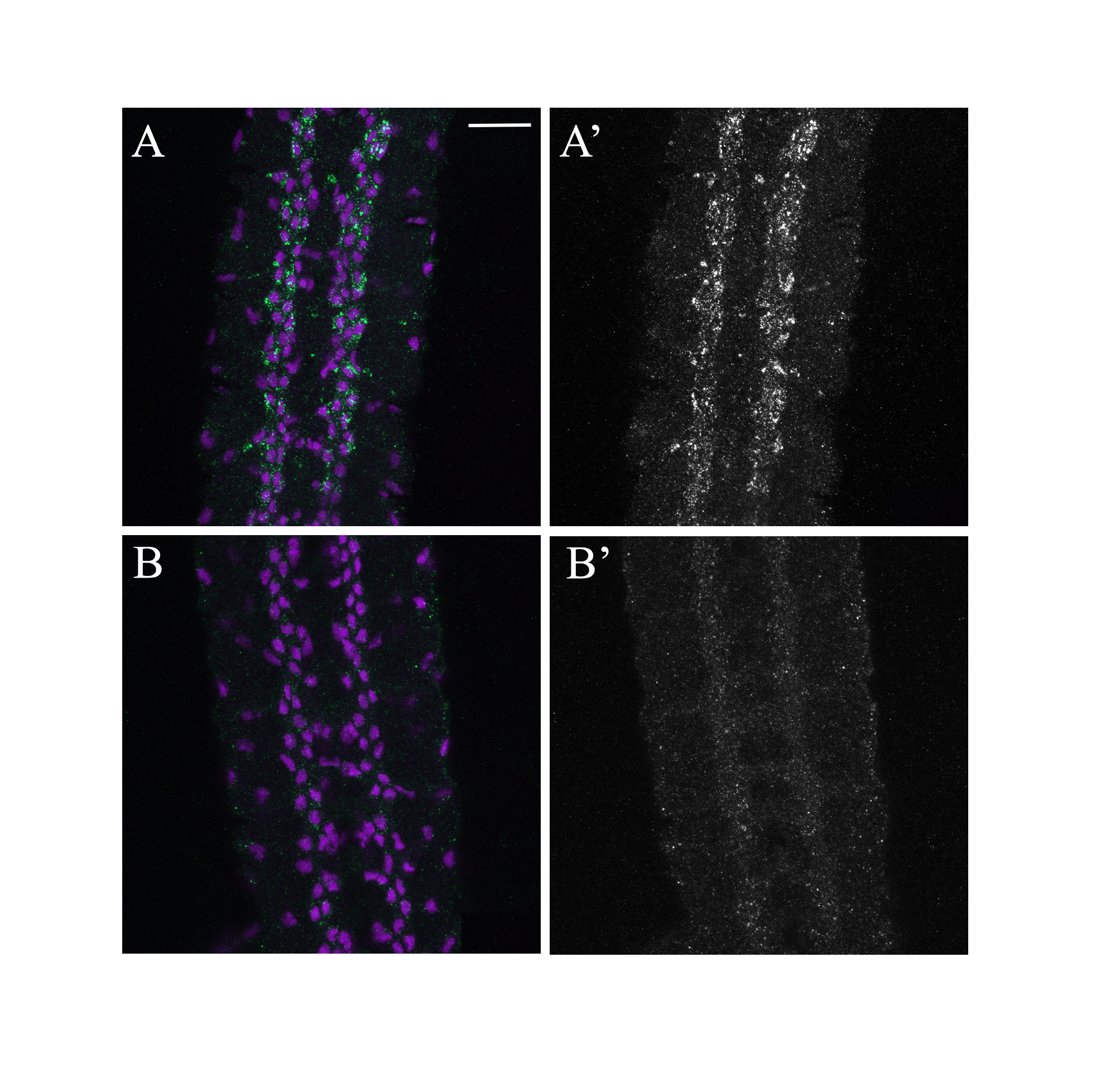

Supplement: jkae032_Supplementary_Data [file jkae032_supplementary_data.zip › Figure_S5_G3-2024-404877.tif]

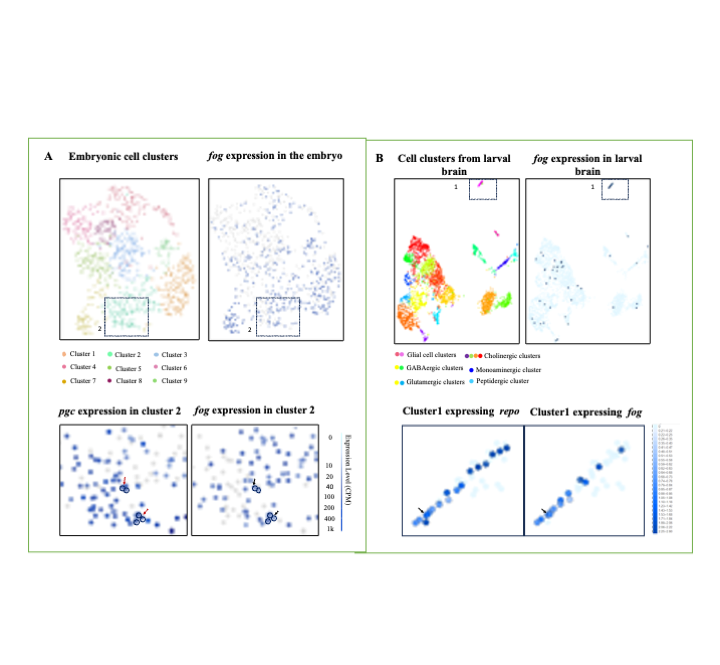

Supplement: jkae032_Supplementary_Data [file jkae032_supplementary_data.zip › Suppl. figure 4-ScRNA seq_250124-Final-Proofed.tiff]
